# Which QT Correction Formulae to Use for QT Monitoring?

**DOI:** 10.1161/JAHA.116.003264

**Published:** 2016-06-17

**Authors:** Bert Vandenberk, Eline Vandael, Tomas Robyns, Joris Vandenberghe, Christophe Garweg, Veerle Foulon, Joris Ector, Rik Willems

**Affiliations:** ^1^ Department of Cardiovascular SciencesUniversity of LeuvenBelgium; ^2^ Department of Pharmaceutical and Pharmacological SciencesUniversity of LeuvenBelgium; ^3^ Department of NeurosciencesUniversity of LeuvenBelgium; ^4^ Department of CardiologyUniversity Hospitals LeuvenLeuvenBelgium

**Keywords:** electrocardiography, mortality, population, QT interval electrocardiography, risk factors, risk prediction, Mortality/Survival, Electrocardiology (ECG), Prognosis, Risk Factors, Electrophysiology

## Abstract

**Background:**

Drug safety precautions recommend monitoring of the corrected QT interval. To determine which QT correction formula to use in an automated QT‐monitoring algorithm in our electronic medical record, we studied rate correction performance of different QT correction formulae and their impact on risk assessment for mortality.

**Methods and Results:**

All electrocardiograms (ECGs) in patients >18 years with sinus rhythm, normal QRS duration and rate <90 beats per minute (bpm) in the University Hospitals of Leuven (Leuven, Belgium) during a 2‐month period were included. QT correction was performed with Bazett, Fridericia, Framingham, Hodges, and Rautaharju formulae. In total, 6609 patients were included (age, 59.8±16.2 years; 53.6% male and heart rate 68.8±10.6 bpm). Optimal rate correction was observed using Fridericia and Framingham; Bazett performed worst. A healthy subset showed 99% upper limits of normal for Bazett above current clinical standards: men 472 ms (95% CI, 464–478 ms) and women 482 ms (95% CI 474–490 ms). Multivariate Cox regression, including age, heart rate, and prolonged QTc, identified Framingham (hazard ratio [HR], 7.31; 95% CI, 4.10–13.05) and Fridericia (HR, 5.95; 95% CI, 3.34–10.60) as significantly better predictors of 30‐day all‐cause mortality than Bazett (HR, 4.49; 95% CI, 2.31–8.74). In a point‐prevalence study with haloperidol, the number of patients classified to be at risk for possibly harmful QT prolongation could be reduced by 50% using optimal QT rate correction.

**Conclusions:**

Fridericia and Framingham correction formulae showed the best rate correction and significantly improved prediction of 30‐day and 1‐year mortality. With current clinical standards, Bazett overestimated the number of patients with potential dangerous QTc prolongation, which could lead to unnecessary safety measurements as withholding the patient of first‐choice medication.

## Introduction

The QT interval is a measure of the duration of ventricular repolarization. It approximates the time interval between the start of depolarization and the end of repolarization of the ventricular myocardium.^1^ QT prolongation is associated with a risk for cardiac arrhythmias given that it can result in early after depolarizations, provoke Torsades des Pointes, and lead to ventricular fibrillation, causing sudden cardiac death.^2^ The risk assessment for QT prolongation of in‐hospital patients is based on their medication profile, demographic risk factors, electrolyte disturbances, and monitoring of the corrected QT (QTc) interval.^3^, ^4^ Large population studies have shown a relation between QTc and all‐cause mortality, cardiac mortality, and sudden cardiac death.^5^, ^6^, ^7^

For correct interpretation, the QT interval should undergo adequate rate correction (QTc) to compare measurements at different time points and at different heart rates. To perform optimal risk stratification, this rate correction has to be reliable. The current clinical standard is the most widely used Bazett formula, but with this formula, there is a known overcorrection at high heart rates and undercorrection at lower heart rates.^8^

In developing an automated patient‐specific drug safety algorithm applicable in the electronic medical record, QTc together with clinical, biochemical, and pharmacological risk factors should be included. We wanted to determine which QT correction formula would be the best to use in such an automated algorithm. We first compared the heart rate correction performance of 5 commonly studied QT correction formulae. Second, we aimed to study the impact of the implementation of different QT correction formulae on risk assessment for mortality. To rule out a potential effect of the normal range or reference value for QTc in our population, we determined the reference values of the different formula in a healthy subset. We used this reference value to study the risk stratification for mortality for each QT correction formula separately. Finally, we investigated whether implementation of these different formulae would influence clinical decision making in a retrospective analysis of patients receiving haloperidol.

## Methods

### Study Population

All electrocardiograms (ECGs) taken in patients ≥18 years of age at the University Hospitals of Leuven (Leuven, Belgium) during a 2‐month period (January 1, 2014 until February 28, 2014) were collected retrospectively using the MUSE Cardiology Information System (GE Medical Systems, Menomonee Falls, WI). All ECGs were standard 12‐lead resting ECGs (25 mm/s paper speed, 10 mm/mV amplitude, and 250 Hz sampling rate). ECGs were recorded using MAC 5500 of GE Healthcare with automated analysis by the “GE Marquette 12SL ECG Analysis Program.” Age, sex, contact type (emergency department, ambulatory, or hospitalized), heart rate, RR interval, QRS duration (QRSd), and QT interval were registered. On May 19, 2015, patients’ mortality status was checked using the electronic medical records determining 30‐day and 1‐year all‐cause mortality.

ECGs were inspected visually for quality, rejecting ECGs with missing leads and excessive noise interfering with analysis. Next, for every patient, only the first ECG was retained and the underlying rhythm was visually controlled.

Patients were excluded from further analysis when not in sinus rhythm, a QRS duration >120 ms, or when the heart rate was >90 beats per minute (bpm). The limitation on heart rate was introduced to minimize overcorrection when correcting the QT interval for RR in case of tachycardia and thereby minimizing outliers influencing further statistics.

The study was approved by the University Hospitals of Leuven ethical committee and obtaining informed consent was waived.

### QT Correction

QT correction for heart rate was performed using 5 previously published formulae:

1. Bazett^9^: QTcB=QT/RR^1/2^
2. Fridericia^10^: QTcFri=QT/RR^1/3^
3. Framingham^11^: QTcFra=QT+0.154 (1−RR)
4. Hodges^12^: QTcH=QT+0.00175 ([60/RR]−60)
5. Rautaharju^13^: QTcR=QT−0.185 (RR−1)+k (k=+0.006 seconds for men and +0 seconds for women)

The Hodges correction formula was originally developed based on heart rate; however, for uniform interpretation, all formulas above are shown for calculations based on QT and RR interval measured in seconds. However, values presented in tables and figures are expressed in milliseconds (ms) conform to clinical use.

Using a population‐based approach, the relation between QTc and RR was determined using scatterplots for QTc/RR pairs per subject. QTc/RR linear regression slopes were calculated with the template: QTc=BxRR+intercept. Given that optimal QTc correction should be independent of the RR interval, the slope of the linear regression (B) and *R* ^2^ should be zero. Therefore, the best performing QT correction formula has a slope value and *R* ^2^ closest to zero, indicating the least remaining influence by the RR interval. However, the true relationship between QTc and RR cannot be exactly linear for all correction formulae simultaneously, but linear QTc/RR regression is a commonly used technique providing a good estimate of remaining influence of the RR interval on the corrected QT values.^14^, ^15^

### Reference Interval

From this cohort, an age‐ and sex‐matched population of 212 healthy patients, ranging from 18 to 70 years of age, was identified. Selection was performed blinded of ECG measurements such as heart rate and QTc. Patients were selected to fulfill the following criteria:

1. No cardiovascular medical history
2. No significant medical history, such as diabetes mellitus, chronic obstructive pulmonary disease, etc.
3. No cardiovascular medication
4. No QT or repolarization influencing medication

Based on this healthy subset, the 99% reference interval, defined as lower and upper limit of normal (LLN and ULN, respectively) and their 90% CI, were calculated for each QT correction formula using the most recent guidelines of the Clinical and Laboratory Standards Institute.^16^

### Retrospective Clinical Analysis

Data from a point‐prevalence study on the use of haloperidol,^17^ a known QT‐prolonging drug with associated risk on Torsade des Pointes, in the University Hospitals of Leuven, were used to perform a clinical simulation of in‐hospital risk stratification based on the previously described 5 correction formulae for QTc. ECGs performed before the administration of a first dose of haloperidol were collected, together with clinical variables. Patients were excluded for further analysis using identical exclusion criteria as stated above. Risk stratification was performed based on clinical standards, 450 and 470 ms cutoffs for men and women, respectively. The number of patients identified above clinical standards was compared between the different correction formulae. The study was approved by the ethical committee of the University Hospitals of Leuven in a separate protocol.

### Statistical Analysis

All continuous variables are given as mean±SD and proportions as percentages. Means were compared using Student *t* tests and proportions using chi‐squared analysis. QTc values between correction formulae were compared using repeated‐measurements 1‐way ANOVA followed by pair‐wise comparison with Tukey correction. QTc/RR linear regression was performed calculating the slope (B value) and the intercept with their 95% CI. Comparison of slopes between correction formulas was performed using 1‐way ANOVA with Tukey correction for multiple comparisons based on the slope, SE of the slope, and the number of comparisons. Bland–Altman plots with calculation of bias and limits of agreement were performed to illustrate differences between correction formulae. For each QT correction formula, the sensitivity, specificity, and positive (PPV) and negative predictive value (NPV) for predicting 30‐day all‐cause mortality were calculated. Multivariate Cox regression analysis, using the ENTER method, for prediction of 30‐day and 1‐year mortality, was performed separately for each QT correction formula for significant univariate parameters. Interpretation of the models was performed by analyzing the log likelihood ratio (−2 LLR) results by analyzing the difference in −2 LLR with QTcB using chi‐squared analysis. *P*<0.05 was considered significant. All statistical analysis was performed using SPSS (IBM Statistics, version 22; IBM Corp, Armonk, NY) and GraphPad (Prism, version 6; GraphPad Software Inc., La Jolla, CA).

## Results

### Demographics

A total of 6609 patients were included in the analysis. Demographics are shown in Table 1. There was a nearly equal distribution of sex (53.6% male vs 46.4% female). Female patients were significantly older, had a higher heart rate, and a shorter QRS duration. There was no sex difference in uncorrected QT interval, but for all QT correction formulae, QTc was significantly longer in females. Results of the QTc values comparison between correction formulae are shown in Table 2. QTcB was significantly longer than all other correction formulae (*P*≤0.0001); only QTcFri and QTcFra did not differ significantly (*P*>0.050).

**Table 1:** Demographics

	All Patients	Male	Female	*P* Value
n (%)	6609	3542 (53.6)	3067 (46.4)	
Ambulatory	3993 (60.4)	2128 (53.3)	1965 (46.7)	0.831
Emergency	1195 (18.1)	645 (54.0)	550 (46.0)	
Hospitalized	1421 (21.5)	769 (54.1)	652 (45.9)	
30‐day mortality (%)	61 (0.9)	31 (50.8)	30 (49.2)	
SCD	1 (1.6)	1 (100)	0 (0)	0.501
Cardiac	20 (32.8)	8 (40)	12 (60)	
Noncardiac	37 (60.7)	20 (54.1)	17 (45.9)	
Unknown	3 (4.9)	2 (66.7)	1 (33.3)	
1‐year mortality (%)	264 (4.0)	144 (54.5)	120 (45.5)	
SCD	8 (3.0)	5 (62.5)	3 (37.5)	0.816
Cardiac	50 (18.9)	26 (52.0)	24 (48.0)	
Noncardiac	158 (59.9)	89 (56.3)	69 (43.7)	
Unknown	48 (18.2)	24 (50.0)	24 (50.0)	
Age, y	59.8±16.2	59.4±15.6	60.3±16.8	0.015
Heart rate, bpm	68.8±10.6	67.5±11.0	70.2±9.9	<0.001
QRS duration, ms	92±11	96±11	87±10	<0.001
QT interval, ms	398±33	398±33	398±32	0.816
QTcB, ms	423±27	419±27	428±27	<0.001
QTcFri, ms	414±25	412±24	417±25	<0.001
QTcFra, ms	414±24	411±24	418±24	<0.001
QTcH, ms	413±24	411±24	416±25	<0.001
QTcR, ms	421±24	420±24	422±24	0.013

bpm indicates beats per minute; QTcB, QT correction with Bazett formula; QTcFra, QT correction with Framingham formula; QTcFri, QT correction with Fridericia formula; QTcH, QT correction with Hodges formula; QTcR, QT correction with Rautaharju formula; SCD, sudden cardiac death.

**Table 2:** Results of the QTc Values Comparison Between Correction Formulae

Comparison	Mean Difference (ms)	95% CI (ms)	*P* Value
QTcB vs QTcFri	8.784	8.410 to 9.157	≤0.0001
QTcB vs QTcFra	8.750	8.404 to 9.096	≤0.0001
QTcB vs QTcH	9.637	9.161 to 10.110	≤0.0001
QTcB vs QTcR	2.270	2.026 to 2.513	≤0.0001
QTcFri vs QTcFra	−0.034	−0.118 to 0.051	>0.050
QTcFri vs QTcH	0.853	0.719 to 0.987	≤0.0001
QTcFri vs QTcR	−6.514	−6.735 to −6.294	≤0.0001
QTcFra vs QTcH	0.887	0.698 to 1.075	≤0.0001
QTcFra vs QTcR	−6.481	−6.651 to −6.311	≤0.0001
QTcH vs QTcR	−7.367	−7.688 to −7.047	≤0.0001

QTcB indicates QT correction with Bazett formula; QTcFra, QT correction with Framingham formula; QTcFri, QT correction with Fridericia formula; QTcH, QT correction with Hodges formula; QTcR, QT correction with Rautaharju formula.

### QTc/RR Analysis

**Figure 1:**
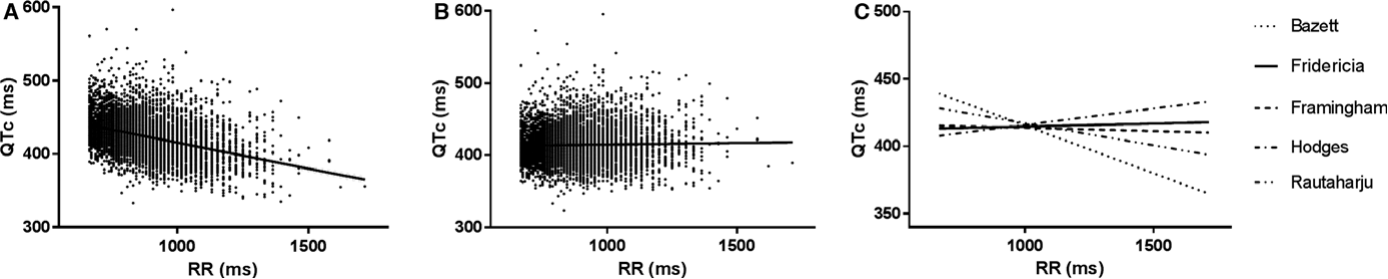
A, QTcB/RR plot and linear regression slope. B, QTcFri/RR plot and linear regression slope. C, Comparison of the linear regression slopes of the different correction formulae. QTc indicates corrected QT interval; RR, QTcB, QT correction with Bazett formula; QTcFri, QT correction with Fridericia formula; RR interval.

**Figure 2:**
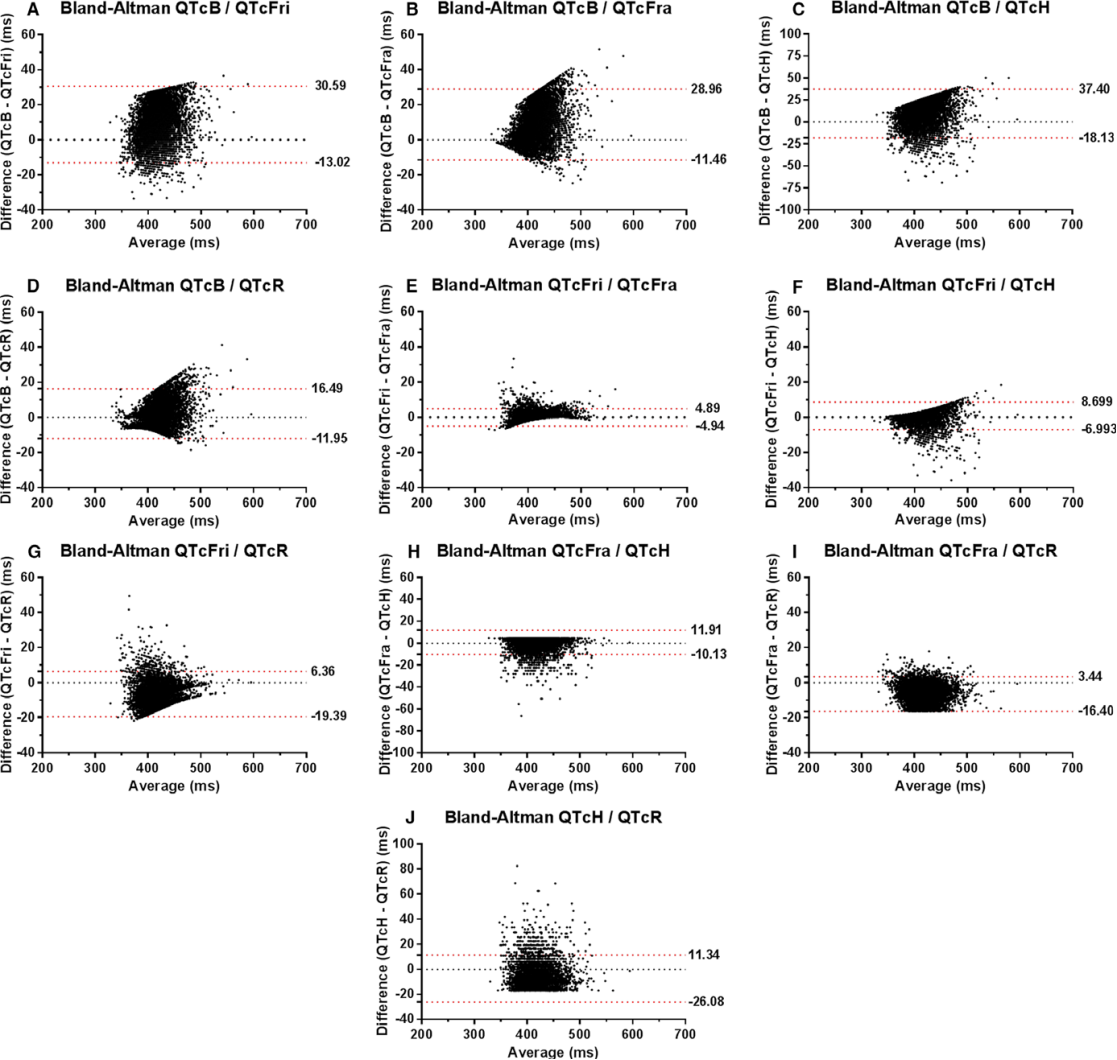
Bland–Altman graphs of the difference versus average of pair‐wise QT correction formulae comparison. The limits of agreement are shown as a dashed red line accompanied by their value. A, Bland–Altman analysis for comparison of QTcB and QTcFri. B, Bland–Altman analysis for comparison of QTcB and QTcFra. C, Bland–Altman analysis for comparison of QTcB and QTcH. D, Bland–Altman analysis for comparison of QTcB and QTcR. E, Bland–Altman analysis for comparison of QTcFri and QTcFra. F, Bland–Altman analysis for comparison of QTcFri and QTcH. G, Bland–Altman analysis for comparison of QTcFri and QTcR. H, Bland–Altman analysis for comparison of QTcFra and QTcH. I, Bland–Altman analysis for comparison of QTcFra and QTcR. J, Bland–Altman analysis for comparison of QTcH and QTcR. QTcB indicates QT correction with Bazett formula; QTcFra, QT correction with Framingham formula; QTcFri, QT correction with Fridericia formula; QTcH, QT correction with Hodges formula; QTcR, QT correction with Rautaharju formula.

The QTc/RR analysis identified the Fridericia and Framingham correction formulae as the best rate correction in this population, with slopes of 0.004 and −0.005, respectively (shown in Table 3 and Figure 1). All slopes of the correction formulae differed significantly from one another, as summarized in Table 4. Bazett's correction formula performed worst, with a slope of −0.071, indicating significant over‐ and underestimation of QTc at high or low heart rates, respectively. Bland–Altman plots and data on the bias and limits of agreement between correction formulae are available as Table 5 and Figure 2. This is illustrated when selecting patients at the extremities of the heart rate spectrum. Selecting the 1190 patients (18.0%) with a heart rate ≥80 bpm (49.0% male; age, 57.7±16.6 years; QRSd, 89±11 ms; heart rate, 84.5±3.2 bpm), the difference between QTcB and QTcFri is 24 ms (QTcB 438 ms and QTcFri 414 ms; *P*<0.001).

**Table 3:** Results QTc/RR Analysis

	Slope	95% CI Slope	Constant (ms)	95% CI Constant (ms)	*R* ^2^
QTcB	−0.071	−0.075 to −0.067	487	483 to 490	0.1438
QTcFri	0.004	<0.001 to 0.008	410	407 to 414	0.0007
QTcFra	−0.005	−0.009 to −0.001	419	415 to 422	0.0009
QTcH	0.024	0.020 to 0.028	392	388 to 395	0.0212
QTcR	−0.033	−0.037 to −0.029	450	447 to 454	0.0390

QTcB indicates QT correction with Bazett formula; QTcFra, QT correction with Framingham formula; QTcFri, QT correction with Fridericia formula; QTcH, QT correction with Hodges formula; QTcR, QT correction with Rautaharju formula; RR, RR interval.

**Table 4:** Comparison of Slopes Between Correction Formulae

Comparison	Mean Difference	95% CI	*P* Value
QTcB vs QTcFri	−0.075	−0.083 to −0.067	≤0.0001
QTcB vs QTcFra	−0.066	−0.074 to −0.058	≤0.0001
QTcB vs QTcH	−0.095	−0.103 to −0.087	≤0.0001
QTcB vs QTcR	−0.038	−0.046 to −0.030	≤0.0001
QTcFri vs QTcFra	0.009	0.001 to 0.017	≤0.050
QTcFri vs QTcH	−0.02	−0.028 to −0.012	≤0.0001
QTcFri vs QTcR	0.037	0.029 to 0.045	≤0.0001
QTcFra vs QTcH	−0.029	−0.037 to −0.021	≤0.0001
QTcFra vs QTcR	0.028	0.020 to 0.036	≤0.0001
QTcH vs QTcR	0.057	0.049 to 0.065	≤0.0001

QTcB, QT correction with Bazett formula; QTcFra, QT correction with Framingham formula; QTcFri, QT correction with Fridericia formula; QTcH, QT correction with Hodges formula; QTcR, QT correction with Rautaharju formula.

**Table 5:** Results of Bland–Altman Analysis Between QT Correction Formulae

Comparison	Bias (ms)	SD of Bias (ms)	95% Limits of Agreement (ms)
QTcB vs QTcFri	8.784	11.125	−13.021 to 30.589
QTcB vs QTcFra	8.750	10.311	−11.460 to 28.961
QTcB vs QTcH	9.637	14.167	−18.130 to 37.404
QTcB vs QTcR	2.270	7.254	−11.948 to 16.487
QTcFri vs QTcFra	−0.033	2.510	−4.954 to 4.887
QTcFri vs QTcH	0.853	4.003	−6.993 to 8.699
QTcFri vs QTcR	−6.514	5.567	−19.385 to 6.356
QTcFra vs QTcH	0.886	5.623	−10.134 to 11.907
QTcFra vs QTcR	−6.481	5.060	−16.398 to 3.437
QTcH vs QTcR	−7.367	9.547	−26.078 to 11.344

QTcB indicates QT correction with Bazett formula; QTcFra, QT correction with Framingham formula; QTcFri, QT correction with Fridericia formula; QTcH, QT correction with Hodges formula; QTcR, QT correction with Rautaharju formula.

### Reference Interval

The reference intervals, LLN and ULN, of the healthy subset are shown with their 90% CIs in Table 6. For LLN, there was only a limited difference for the different QT correction formulae, and sex did not seem to affect the LLN value. For the ULN, there were, as expected, a difference between males and females. Bazett had the highest ULN values in both sexes, up to 472 ms for men and 482 ms for women. The QT correction formulae with the best rate adaptation, Fridericia and Framingham, produced an ULN similar to current clinical values.

**Table 6:** Reference Interval by Sex

	LLN	90% CI LLN	ULN	90% CI ULN
Male				
QTcB, ms	346	338 to 355	472	464 to 478
QtcFri, ms	349	343 to 356	448	442 to 454
QtcFra, ms	350	344 to 357	449	443 to 455
QTcH, ms	351	345 to 357	446	440 to 452
QTcR, ms	356	349 to 364	464	457 to 470
Female				
QTcB, ms	355	348 to 364	482	474 to 490
QTcFri, ms	348	341 to 355	468	460 to 476
QTcFra, ms	351	344 to 358	467	458 to 474
QTcH, ms	348	341 to 355	465	457 to 473
QTcR, ms	356	349 to 363	470	462 to 477

Reference interval for each correction formula based on the healthy population divided by gender. LLN, lower limit of normal; QTcB, QT correction with Bazett formula; QTcFra, QT correction with Framingham formula; QTcFri, QT correction with Fridericia formula; QTcH, QT correction with Hodges formula; QTcR, QT correction with Rautaharju formula; ULN, upper limit of normal.

The number of patients with a QTc below LLN was limited: from 5 patients (0.07%) for QTcFri and QTcH to 12 patients (0.18%) using QTcFra. The correction formulae with a more‐negative slope identified more patients as having a short QTc, using QTcB 14 patients (0.21%) and for QTcR 26 patients (0.39%). Table 7 shows the proportion of patients with a QTc higher than the ULN.

**Table 7:** Patients With QTc>ULN and All‐Cause Mortality Risk Stratification

	QTc>ULN	30‐Day				1‐Year			
		Sens	Spec	PPV	NPV	Sens	Spec	PPV	NPV
QTcB	3.2%	19.7%	97.0%	5.7%	99.2%	12.1%	97.2%	15.2%	96.4%
QTcFri	5.2%	27.9%	95.0%	4.9%	99.3%	16.3%	95.3%	12.5%	96.5%
QTcFra	4.6%	27.9%	95.6%	5.5%	99.3%	14.1%	95.7%	12.1%	96.4%
QTcH	5.7%	26.2%	94.5%	4.3%	99.3%	14.8%	94.7%	10.4%	96.4%
QTcR	3.5%	23.0%	96.7%	6.0%	99.3%	12.5%	96.9%	14.2%	96.4%

NPV indicates negative predictive value; PPV, positive predictive value; QTcB, QT correction with Bazett formula; QTcFra, QT correction with Framingham formula; QTcFri, QT correction with Fridericia formula; QTcH, QT correction with Hodges formula; QTcR, QT correction with Rautaharju formula; Sens, sensitivity; Spec, specificity; ULN, upper limit of normal.

### Mortality Analysis

In total, 0.9% of the patients died at 30 days and 4.0% at 1 year. The 30‐day mortality did not differ significantly for contact type (emergency department, ambulatory, or hospitalized; *P*=0.224). However, 1‐year mortality was more frequent (*P*<0.001) in hospitalized patients (6.0%) than ambulatory (3.8%) and patients presenting at the emergency department (2.7%).

In Table 7, the sensitivity, specificity, PPV, and NPV for mortality prediction for QTc>ULN are shown. There were no deaths occurring in patients with a QTc<LLN. At 30 days, QTcB had a far lower sensitivity with equal specificity compared to the best performing correction formulae. After 1 year, the differences became less, but remained consistent.

The Cox regression analysis is summarized in Table 8; the complete results are available in Tables S1 through S4. The 30‐day mortality model included age, heart rate, and a QTc>ULN as univariate significant predictors. Multivariate analysis identified age, heart rate, and QTc>ULN as independent predictors of 30‐day all‐cause mortality. Comparing the LLR analysis, all QTc formulae were significantly better predictors, compared to QTcB (QTcFri, *P*<0.005; QTcH, *P*<0.005; QTcR, *P*<0.010), with the model containing QTcFra (*P*<0.001) performing the best. The model with QTcFra was also significant better than these with QTcH and QTcR (*P*<0.050), but did not differ significantly with QTcFri.

**Table 8:** Results for QTc>ULN in Multivariate Cox Regression Analysis

	30‐Day All‐Cause Mortality				1‐Year All‐Cause Mortality			
	HR	95% CI	*P* Value	−2 LLR ΔQTcB	HR	95% CI	*P* Value	−2 LLR ΔQTcB
QTcB	4.49	2.31 to 8.74	<0.001	0	2.90	1.97 to 4.27	<0.001	0
QTcFri	5.95	3.34 to 10.60	<0.001	12.73‡	3.24	2.31 to 4.54	<0.001	13.32‡
QTcFra	7.31	4.10 to 13.05	<0.001	18.09§	3.20	2.23 to 4.58	<0.001	8.21*
QTcH	6.18	3.41 to 11.20	<0.001	11.94‡	3.04	2.13 to 4.34	<0.001	6.87
QTcR	6.32	3.43 to 11.64	<0.001	9.97†	3.32	2.29 to 4.82	<0.001	7.10

Level of significance: For 30‐day all‐cause mortality analysis, a ΔLLR between models >5.99 is statistically significant at level *P*<0.050. For all QTc formulae, the regression models were significantly better compared to QTcB. The model with QTcFra was significantly better than those with QTcH and QTcR. For 1‐year all‐cause mortality analysis, a ΔLLR between models >7.81 is statistically significant at level *P*<0.050. The regression models with QTcFri and QTcFra were significantly better predictors compared to QTcB. There were no other significant differences between QTc formulae. −2 LLR Δ QTcB indicates difference of −2 log likelihood ratio compared to the model including QTcB; HR, hazard ratio; QTcB, QT correction with Bazett formula; QTcFra, QT correction with Framingham formula; QTcFri, QT correction with Fridericia formula; QTcH, QT correction with Hodges formula; QTcR, QT correction with Rautaharju formula.

*P<0.050; ^†^ *P*<0.010; ^‡^ *P*<0.005; ^§^ *P*<0.001.

The 1‐year mortality model also included QRSd as a univariate significant predictor. Age, heart rate, and QTc>ULN (and QRSd in the model with QTcFri) were identified as independent predictors. The models with QTcFri and QTcFra remained significant better predictors compared to QTcB (QTcFra, *P*<0.050; QTcFri, *P*<0.005). All other models did not differ significantly from one another.

Subgroup analysis for cause of death was performed at 1‐year given the low number of events at 30 days (available in Tables S1 through S4). For cardiac mortality, a longer QRS duration, even in a population with QRS <120 ms, was an independent predictor of mortality, and the models did not differ significantly for QT correction formulae. Whereas for noncardiac mortality, the opposite was true and a shorter QRS duration seemed an independent predictor of noncardiac mortality and models with QTcFri and QTcH were significantly better predictors. Comparison of QRSd between patients from the healthy cohort (88.13±11.21 ms), with these dying from cardiac causes (94.88±12.39 ms; *P*<0.001) and noncardiac causes (88.66±11.82 ms; *P*=0.663), showed that the QRSd in patients who died of noncardiac causes was not shorter than normal. Taken together, these data showed that, in our hospital, population QRS duration was related with a worse cardiovascular prognosis. Patients with a “broader” QRS complex have a higher mortality and die more often of cardiac causes than those with a more “narrow” QRS complex, who have a better survival, and if they die, this was caused more often by noncardiac causes.

### Retrospective Clinical Analysis

In total, 212 patients were included in the haloperidol point‐prevalence study. Mean age of the patients was 73±15 years and 127 (59.9%) were male. Sinus rhythm was present in 167 (78.8%), 39 (18.4%) were in atrial fibrillation, 6 (2.8%) had ventricular pacemaker rhythm, and 31 (14.6%) had a QRS >120 ms. After selecting patients, as stated in the exclusion criteria, 107 (50.5%) were eligible for further analysis.

Use of QTcB in clinical routine identified 33 patients (30.8%) with QTcB values above clinical standard, hence deemed at risk for a possible clinical effect of the prescribed drug by exaggerated QT prolongation. Use of QTcFri or QTcFra identified only 20 (18.7%) or 16 (15.0%) patients above clinical cut‐off values for QTc. Use of these correction formulae would reduce significantly (*P*<0.001) the number of patients identified as at possible risk for Torsade de Pointes by dangerous QT prolongation. This could have possible implications on clinical decision making in these patients.

## Discussion

This analysis confirms the inferiority of QTcB for rate correction compared to QTcFri or QTcFra, even in patients in sinus rhythm and normal heart rate ranges. In a healthy subset of our study population, this inferior rate correction of QTcB led to the calculation of reference values with a ULN far higher than current clinical standards, whereas formulae with near optimal rate correction approximate the current clinical standards for both males and females. Moreover, QTcFri and QTcFra showed to be better predictors of 30‐day and 1‐year all‐cause mortality.

### QT Correction

The inferiority of QTcB has already been widely documented in smaller population studies.^15^, ^18^ Also, alternative approaches in which the QT correction formula was chosen based on sex and heart rate range have been suggested.^19^ Within different populations, it can be expected that certain formulae perform better in rate correction, which could be based on sex composition, ethnicity, age, exclusion of higher or lower heart rates, etc. Sex analysis in our population confirmed the difference in rate correction between male and females for different QT correction formulae. The largest differences were observed with QTcFri (slope, 0.002 vs 0.016 in males vs females, respectively) and QTcFra (slope, −0.010 vs 0.011 in male vs females, respectively). The switch from a negative to a positive slope observed in QTcFra indicates a switch from a slight overcorrection in males toward a slight undercorrection in females at higher heart rates. Notwithstanding these changes, QTcFri and QTcFra remained the best performing correction formulae for both sexes.

Thus, different studies have suggested different formulae to be superior, but all had one thing in common: the inferiority of QTcB.^8^, ^15^, ^19^, ^20^ The introduced difference by using QTcB instead of a superior rate‐correcting formula might be clinically significant. We observed an overcorrection with a mean difference of 24 ms between QTcB and QTcFri in patients with a heart rate ranging 80 to 90 bpm. This difference could lead to changes in clinical practice, for example, withholding a patient from clinically indicated first‐choice medication. If we keep in mind that thorough QT/QTc studies should be designed to detect QTc changes of 5 ms, the observed difference between formulae should lead to a reconsideration of current clinical practice in which QTcB is still the most used formula.^21^

Besides a population‐based approach and choosing the best performing formula within this population, an individual QT correction could be obtained. It was stated by Malik et al. that a mathematical QT/RR relation that fits for all persons individually is unobtainable.^22^ Individual‐ or subject‐specific correction methods have proven to be superior to population‐based formulas.^8^, ^22^, ^23^ However, the reliability of a subject‐specific correction depends on the number of data points, the range of heart rates between different data points, and the time interval between data points and possible intervening changes in between, such as medication, physical activity, and the interplay between sympathetic and parasympathetic tone. Adding the recently described nonlinearity of subject‐specific QT/RR relations and the problem of QT hysteresis makes the subject‐specific QT correction currently rather a scientific tool suited for thorough QT/QTc studies and further study of the QT/RR relation, rather than for routine clinical analysis.^24^

### Reference Interval

The determined LLN within the healthy subpopulation corresponded to the proposed lower limit of 350 ms, with a maximal variation of only 6 ms for both men and women for all QT correction formulae studied.^25^ This is in contrast to the ULN, where approximation of the clinical standard of 450 ms for QTcFri (448 ms), QTcFra (449 ms), and QTcH (446 ms) was observed. However, correction formulae with more‐negative slopes, QTcB (472 ms) and QTcR (464 ms), have far higher ULNs than the clinical standard. Again, this indicates the impact of suboptimal rate correction, even in a healthy population. In women, the ULN approximates the clinical standard of 470 ms, except for QTcB with 482 ms.

A comparable analysis was performed by Luo et al. (n=10 303) excluding only the top 2% of QTc values.^19^ They reported a 2% ULN within heart rate ranges of 60 to 90 bpm of 480 ms in men and 486 ms in women for QTcB, whereas the other reported formulae approximated the clinical standards.

A careful clinical interpretation of these findings suggest that using the clinical standards of 450 ms in men and 470 ms in women leads to an overestimation of patients with prolonged QT when using QTcB. Applying these clinical standards in our population with QTcB would triple the proportion of patients with a QTc>ULN from 3.2% to 9.4%. This in contrast to the 4.6% using QTcFra, which is still twice the amount of patients requiring attention for a prolonged QTc.

### Prediction of Mortality

We report that a prolonged QTc is related to an increased 30‐day and 1‐year mortality and that the use of QTcFri or QTcFra would significantly improve all‐cause mortality risk stratification compared to QTcB. A prolonged QRSd is related to 1‐year mortality. We hypothesize that the apparent association between a shorter QRSd and a higher risk of noncardiac mortality is an illustration of the concept of conversion of mode of death (a decrease of cardiovascular mortality leads to an increase in noncardiovascular death) within this hospital‐based population, but should be interpreted with caution because this finding could be influenced by our design and the study limitations.

Several population‐based studies have previously reported an association between QTc and all‐cause mortality. The Framingham Heart Study population (n=6895; mean follow‐up=27.5 years) observed a significant relation between every 20‐ms increase in QTcB and all‐cause mortality (hazard ratio [HR], 1.14; 95% CI, 1.10–1.18; *P*<0.0001), coronary heart disease–related mortality (HR, 1.15; 95% CI, 1.05–1.26; *P*=0.003), and sudden cardiac death (HR, 1.19; 95% CI, 1.03–1.37; *P*=0.02).^5^ However, using QTcFra, the formula based on a subset of this study population and therefore superior to QTcB, these findings were less strong. The significant relation with all‐cause mortality remained (HR, 1.09; 95% CI, 1.04–1.13; *P*<0.0001); however, coronary heart disease–related mortality (HR, 1.07; 95% CI, 0.96–1.20; *P*=0.22) and sudden cardiac death (HR, 1.16; 95% CI, 0.96–1.40; *P*=0.12) were no longer significantly associated with the corrected QT interval. Analyzing the data as the risk associated with a prolonged QTcB (450 ms for male, 470 ms for females), there was a significant relation with all‐cause mortality (HR, 1.84; 95% CI, 1.36–2.49; *P*<0.0001) and coronary heart disease mortality (HR, 2.63; 95% CI, 1.36–5.11; *P*=0.004), but not with sudden cardiac death (HR, 1.83; 95% CI, 0.56–5.91; *P*=0.31). Similar analysis with QTcFra was not reported.

In a healthy population study by Schouten et al., a QTcB above 440 ms was associated with a significant relative risk of 1.8 for 15‐year all‐cause mortality in both males and females.^6^ Ischemic heart disease–related 15‐year mortality yielded a significant relative risk of 2.2 in males, but was not significant in females (relative risk, 1.1).

A large primary care population ages between 50 and 90 years (n=173 529; mean follow‐up, 6.1 years) was studied by Nielsen et al., who used QTcFra percentiles and their association with mortality.^7^ In women, a QTcFra ≥470 ms was significantly associated with all‐cause mortality (HR, 1.52; 95% CI, 1.35–1.71), cardiovascular mortality (HR, 2.09; 95% CI, 1.69–2.58), and noncardiovascular mortality (HR, 1.38; 95% CI, 1.19–1.59). In men, a QTcFra ≥466 ms was significantly associated with all‐cause mortality (HR, 2.53; 95% CI, 2.15–2.98), cardiovascular mortality (HR, 4.08; 95% CI, 2.93–5.69), and noncardiovascular mortality (HR, 2.14; 95% CI, 1.77–2.60).

The major difference with the previous studies of the Framingham Heart Study population and the study by Schouten et al. is that our analysis was performed on a hospital‐based population, which cannot be interpreted as a healthy or normal population. Also, these studies reported long‐term follow‐up, and only the Framingham Heart Study reported both QTcB and QTcFra, but still showed a significant association with all‐cause mortality. The primary care study by Nielsen et al. reported only a population ages between 50 and 90 years of age and therefore could have shown higher HR. The high HR of our analysis could be caused by the fact that we studied a hospital‐based nonhealthy population, including 21.5% hospitalized patients, and by the fact that we analyzed 30‐day and 1‐year mortality instead of multiple years of follow‐up.

### Retrospective Clinical Analysis

Haloperidol should not be used in patients with QT prolongation. The data from the haloperidol point‐prevalence study illustrated that when using optimal QT correction, the proportion of patients with QTc values above clinical standards before the prescription of this QT‐prolonging drug could be reduced with up to 50%. This implies that a hospital‐wide automated algorithm, which uses QTcB for assessment of a possible dangerous QT prolongation, would generate double the amount of alerts compared to an algorithm using the optimal QT correction formula. This could lead to alert fatigue and avoiding clinically indicated first‐choice drugs because of potential QT‐prolonging effects.^26^ Hence, using an optimal QT correction formula could reduce the workload and improve patient safety.

### Limitations

One of the main limitations of this study is its single‐center retrospective character. The survival status of the patients was based on the electronic medical record at our institution, without a link to a public register. Although our electronic medical record is linked to multiple regional hospitals, the number of events might be underestimated. We limited current analysis to patients in sinus rhythm with a narrow QRS and a heart rate <90 bpm in order to avoid the number of outliers. The number of parameters included in the risk stratification modeling was limited; however, the data set yielded no missing values. Furthermore, prospective population studies should be used to study population‐based rate correction in abnormal heart rhythms, such as sinus tachycardia, atrial fibrillation, or ventricular conduction defects.

## Conclusion

The current use of Bazett's QT correction formula in clinical standards should be questioned. The use of QTcFri or QTcFra in a hospital‐based population would significantly increase risk stratification for all‐cause mortality. Therefore, the question arises of whether QTcFri, as in thorough QT/QTc studies, should become the next clinical standard replacing QTcB for hospital‐based QT monitoring.

## Disclosures

Willems, Ector, and Garweg have received research funding from Biotronik, Boston Scientific, and Medtronic and speakers and consultancy fees from Biotronik, Boston Scientific, Medtronic, St Jude Medical, and Sorin. Willems and Ector are supported as postdoctoral clinical researcher and Garweg as predoctoral clinical researcher by the Fund for Scientific Research Flanders. Vandael is supported by funding of the Belgian government agency for Innovation by Science and Technology (IWT).

## Supporting information


**Table S1.** Multivariate Cox Regression Analysis for 30‐Day All‐Cause Mortality
**Table S2.** Multivariate Cox Regression Analysis for 1‐Year All‐Cause Mortality
**Table S3.** Multivariate Cox Regression Analysis for 1‐Year Cardiac Mortality
**Table S4.** Multivariate Cox Regression Analysis for 1‐Year Noncardiac Mortality

## References

[1] Garson A Jr . How to measure the QT interval—what is normal? Am J Cardiol. 1993;72:14B–16B. 8256749 10.1016/0002-9149(93)90034-a

[2] van Noord C , Eijgelsheim M , Stricker BH . Drug‐ and non‐drug‐associated QT interval prolongation. Br J Clin Pharmacol. 2010;70:16–23. 20642543 10.1111/j.1365-2125.2010.03660.x PMC2909803

[3] Drew BJ , Ackerman MJ , Funk M , Gibler WB , Kligfield P , Menon V , Philippides GJ , Roden DM , Zareba W ; American Heart Association Acute Cardiac Care Committee of the Council on Clinical Cardiology tCoCN, the American College of Cardiology F . Prevention of torsade de pointes in hospital settings: a scientific statement from the American Heart Association and the American College of Cardiology Foundation. Circulation. 2010;121:1047–1060. 20142454 10.1161/CIRCULATIONAHA.109.192704 PMC3056123

[4] Ray WA , Chung CP , Murray KT , Hall K , Stein CM . Atypical antipsychotic drugs and the risk of sudden cardiac death. N Engl J Med. 2009;360:225–235. 19144938 10.1056/NEJMoa0806994 PMC2713724

[5] Noseworthy PA , Peloso GM , Hwang SJ , Larson MG , Levy D , O'Donnell CJ , Newton‐Cheh C . QT interval and long‐term mortality risk in the Framingham Heart Study. Ann Noninvasive Electrocardiol. 2012;17:340–348. 23094880 10.1111/j.1542-474X.2012.00535.x PMC3481183

[6] Schouten EG , Dekker JM , Meppelink P , Kok FJ , Vandenbroucke JP , Pool J . QT interval prolongation predicts cardiovascular mortality in an apparently healthy population. Circulation. 1991;84:1516–1523. 1914093 10.1161/01.cir.84.4.1516

[7] Nielsen JB , Graff C , Rasmussen PV , Pietersen A , Lind B , Olesen MS , Struijk JJ , Haunso S , Svendsen JH , Kober L , Gerds TA , Holst AG . Risk prediction of cardiovascular death based on the QTc interval: evaluating age and gender differences in a large primary care population. Eur Heart J. 2014;35:1335–1344. 24603310 10.1093/eurheartj/ehu081 PMC4028611

[8] Malik M . Problems of heart rate correction in assessment of drug‐induced QT interval prolongation. J Cardiovasc Electrophysiol. 2001;12:411–420. 11332559 10.1046/j.1540-8167.2001.00411.x

[9] Bazett HC . An analysis of the time‐relations of the electrocardiograms. Heart. 1920;7:353–370.

[10] Fridericia LS . Die systolendauer im elektrokardiogramm bei normalen menschen und bei herzkranken. Acta Med Scand. 1920;53:469–486.

[11] Sagie A , Larson MG , Goldberg RJ , Bengtson JR , Levy D . An improved method for adjusting the QT interval for heart rate (the Framingham Heart Study). Am J Cardiol. 1992;70:797–801. 1519533 10.1016/0002-9149(92)90562-d

[12] Hodges MS , Salerno D , Erlinen D . Bazett's QT correction reviewed: evidence that a linear QT correction for heart rate is better. J Am Coll Cardiol. 1983;1:694.

[13] Rautaharju PM , Zhang ZM , Prineas R , Heiss G . Assessment of prolonged QT and JT intervals in ventricular conduction defects. Am J Cardiol. 2004;93:1017–1021. 15081446 10.1016/j.amjcard.2003.12.055

[14] Dogan A , Tunc E , Varol E , Ozaydin M , Ozturk M . Comparison of the four formulas of adjusting QT interval for the heart rate in the middle‐aged healthy Turkish men. Ann Noninvasive Electrocardiol. 2005;10:134–141. 15842424 10.1111/j.1542-474X.2005.05604.x PMC6932732

[15] Strohmer B , Schernthanere C , Paulweber B , Pichler M . Gender‐specific comparison of five QT correction formulae in middle‐aged participants in an atherosclerosis prevention program. Med Sci Monit. 2007;13:CR165–CR171. 17392645

[16] Horowitz GL . Defining, Establishing, and Verifying Reference Intervals in the Clinical Laboratory; Approved Guideline. 3rd ed Wayne, PA: Clinical and Laboratory Standards Institute; 2008.

[17] Vandael E , Vandenberk B , Vandenberghe J , Spriet I , Willems R , Foulon V . Risk management of QTc‐prolongation in patients receiving haloperidol: an epidemiological study in a University Hospital in Belgium. Int J Clin Pharm. 2016;38:310–320. 26749342 10.1007/s11096-015-0242-9

[18] Puddu PE , Jouve R , Mariotti S , Giampaoli S , Lanti M , Reale A , Menotti A . Evaluation of 10 QT prediction formulas in 881 middle‐aged men from the seven countries study: emphasis on the cubic root Fridericia's equation. J Electrocardiol. 1988;21:219–229. 3171455 10.1016/0022-0736(88)90096-9

[19] Luo S , Michler K , Johnston P , Macfarlane PW . A comparison of commonly used QT correction formulae: the effect of heart rate on the QTc of normal ECGs. J Electrocardiol. 2004;37(suppl):81–90. 15534815 10.1016/j.jelectrocard.2004.08.030

[20] Chiladakis J , Kalogeropoulos A , Arvanitis P , Koutsogiannis N , Zagli F , Alexopoulos D . Heart rate‐dependence of QTc intervals assessed by different correction methods in patients with normal or prolonged repolarization. Pacing Clin Electrophysiol. 2010;33:553–560. 20025715 10.1111/j.1540-8159.2009.02657.x

[21] Darpo B , Nebout T , Sager PT . Clinical evaluation of QT/QTc prolongation and proarrhythmic potential for nonantiarrhythmic drugs: the International Conference on Harmonization of Technical Requirements for Registration of Pharmaceuticals for Human Use E14 guideline. J Clin Pharmacol. 2006;46:498–507. 16638733 10.1177/0091270006286436

[22] Malik M , Farbom P , Batchvarov V , Hnatkova K , Camm AJ . Relation between QT and RR intervals is highly individual among healthy subjects: implications for heart rate correction of the QT interval. Heart. 2002;87:220–228. 11847158 10.1136/heart.87.3.220 PMC1767037

[23] Desai M , Li L , Desta Z , Malik M , Flockhart D . Variability of heart rate correction methods for the QT interval. Br J Clin Pharmacol. 2003;55:511–517. 12814443 10.1046/j.1365-2125.2003.01791.x PMC1884246

[24] Malik M , Hnatkova K , Kowalski D , Keirns JJ , van Gelderen EM . QT/RR curvatures in healthy subjects: sex differences and covariates. Am J Physiol Heart Circ Physiol. 2013;305:H1798–H1806. 24163079 10.1152/ajpheart.00577.2013 PMC3882544

[25] Goldenberg I , Moss AJ , Zareba W . QT interval: how to measure it and what is “normal”. J Cardiovasc Electrophysiol. 2006;17:333–336. 16643414 10.1111/j.1540-8167.2006.00408.x

[26] Beeler PE , Bates DW , Hug BL . Clinical decision support systems. Swiss Med Wkly. 2014;144:w14073. 25668157 10.4414/smw.2014.14073

