# Supplementary material for: Which QT Correction Formulae to Use for QT Monitoring?
Source: J Am Heart Assoc. 2016 Jun 17;5(6):e003264. doi: 10.1161/JAHA.116.003264 (PMC4937268; doi:10.1161/JAHA.116.003264)
Supplement: Supplementary file 1 — Table S1. Multivariate Cox Regression Analysis for 30‐Day All‐Cause Mortality Table S2. Multivariate Cox Regression Analysis for 1‐Year All‐Cause Mortality Table S3. Multivariate Cox Regression Analysis for 1‐Year Cardiac Mortality Table S4. Multivariate Cox Regression Analysis for 1‐Year Noncardiac Mortality [file JAH3-5-e003264-s001.pdf]

SUPPLEMENTAL MATERIAL

Table S1. Multivariate Cox Regression analysis for 30-day all-cause mortality

	Age			Heart rate			QTc>ULN			-2 LLR
	HR	95% CI	p-value	HR	95% CI	p-value	HR	95% CI	p-value	Δ QTcB
QTcB	1.04	1.02 - 1.06	<0.001	1.04	1.01 - 1.06	0.004	4.49	2.31 - 8.74	<0.001	0
QTcFri	1.04	1.02 - 1.06	<0.001	1.05	1.03 - 1.08	<0.001	5.95	3.34 - 10.60	<0.001	12.73†
QTcFra	1.04	1.02 - 1.06	<0.001	1.05	1.03 - 1.08	<0.001	7.31	4.10 - 13.05	<0.001	18.09‡
QTcH	1.04	1.02 - 1.06	<0.001	1.06	1.03 - 1.08	<0.001	6.18	3.41 - 11.20	<0.001	11.94†
QTcR	1.04	1.02 - 1.06	<0.001	1.05	1.02 - 1.07	<0.001	6.32	3.43 - 11.64	<0.001	9.97°

Level of significance: In a 3 parameters analysis a Δ -2 LLR between models >5.99 is statistically significant at level p<0.050. For all QTc formulae the regression models were significant better compared to QTcB. The model with QTcFra was significant better than these with QTcH and QTcR.

° =p<0.010; † =p<0.005; ‡ =p<0.001

Abbreviations:

-2 LLR Δ QTcB: difference of -2 Log Likelihood Ratio compared to the model including QTcB; CI: confidence interval; HR: hazard ratio; QTc>ULN: QTc value above upper limit of normal; QTcB: QT correction with Bazett formula; QTcFri: QT correction with Fridericia formula; QTcFra: QT correction with Framingham formula; QTcH: QT correction with Hodges formula; QTcR: QT correction with Rautaharju formula.

Table S2. Multivariate Cox Regression analysis for 1-year all-cause mortality

	Age			Heart rate			QRSd			QTc>ULN			-2 LLR
	HR	95% CI	p-value	HR	95% CI	p-value	HR	95% CI	p-value	HR	95% CI	p-value	Δ QTcB
QTcB	1.043	1.034-1.053	<0.001	1.030	1.018-1.043	<0.001	0.990	0.980-1.001	0.074	2.899	1.968-4.270	<0.001	0
QTcFri	1.043	1.033-1.052	<0.001	1.037	1.025-1.049	<0.001	0.989	0.979-1.000	0.044	3.238	2.307-4.544	<0.001	13.32†
QTcFra	1.044	1.034-1.053	<0.001	1.038	1.026-1.051	<0.001	0.990	0.979-1.001	0.062	3.197	2.232-4.581	<0.001	8.21*
QTcH	1.044	1.034-1.053	<0.001	1.040	1.028-1.052	<0.001	0.990	0.979-1.000	0.061	3.043	2.132-4.343	<0.001	6.87
QTcR	1.043	1.034-1.053	<0.001	1.035	1.023-1.048	<0.001	0.991	0.980-1.001	0.083	3.321	2.287-4.824	<0.001	7.10

Level of significance: In a 4 parameters analysis a Δ -2 LLR between models >7.81 is statistically significant at level p<0.050. The regression models with QTcFri and QTcFra were significant better predictors of 1-year all-cause mortality compared to QTcB. There were no other significant differences between QTc formulae.

* =p<0.050; † =p<0.005

Abbreviations:

-2 LLR Δ QTcB: difference of -2 Log Likelihood Ratio compared to the model including QTcB; CI: confidence interval; HR: hazard ratio; QTc>ULN: QTc value above upper limit of normal; QTcB: QT correction with Bazett formula; QTcFri: QT correction with Fridericia formula; QTcFra: QT correction with Framingham formula; QTcH: QT correction with Hodges formula; QTcR: QT correction with Rautaharju formula.

Table S3. Multivariate Cox Regression analysis for 1-year cardiac mortality

	Age			Heart rate			QRSd			QTc>ULN			-2 LLR
	HR	95% CI	p-value	HR	95% CI	p-value	HR	95% CI	p-value	HR	95% CI	p-value	Δ QTcB
QTcB	1.042	1.022-1.063	<0.001	1.036	1.010-1.063	0.006	1.023	1.001-1.046	0.039	4.484	2.252-8.930	<0.001	0
QTcFri	1.042	1.022-1.063	<0.001	1.049	1.023-1.075	<0.001	1.023	1.000-1.045	0.047	4.355	2.310-8.213	<0.001	2.04
QTcFra	1.044	1.023-1.064	<0.001	1.051	1.024-1.077	<0.001	1.024	1.002-1.046	0.035	4.258	2.185-8.297	<0.001	-0.27
QTcH	1.045	1.024-1.066	<0.001	1.052	1.027-1.079	<0.001	1.025	1.003-1.048	0.026	3.420	1.713-6.827	<0.001	-4.56
QTcR	1.042	1.022-1.063	<0.001	1.046	1.020-1.072	<0.001	1.023	1.001-1.046	0.039	5.646	2.965-10.752	<0.001	6.19

Level of significance: In a 4 parameters analysis a Δ -2 LLR between models >7.81 is statistically significant at level $p<0.050$. There were no significant differences between models.

Abbreviations:

-2 LLR Δ QTcB: difference of -2 Log Likelihood Ratio compared to the model including QTcB; CI: confidence interval; HR: hazard ratio; QTc>ULN: QTc value above upper limit of normal; QTcB: QT correction with Bazett formula; QTcFri: QT correction with Fridericia formula; QTcFra: QT correction with Framingham formula; QTcH: QT correction with Hodges formula; QTcR: QT correction with Rautaharju formula.

Table S4. Multivariate Cox Regression analysis for 1-year non-cardiac mortality

	Age			Heart rate			QRSd			QTc>ULN			-2 LLR
	HR	95% CI	p-value	HR	95% CI	p-value	HR	95% CI	p-value	HR	95% CI	p-value	Δ QTcB
QTcB	1.045	1.032-1.057	<0.001	1.022	1.006-1.037	0.006	0.979	0.966-0.993	0.003	2.734	1.620-4.613	<0.001	0
QTcFri	1.044	1.031-1.056	<0.001	1.028	1.013-1.044	<0.001	0.977	0.964-0.991	0.001	3.376	2.184-5.219	<0.001	12.10°
QTcFra	1.045	1.032-1.057	<0.001	1.029	1.014-1.045	<0.001	0.978	0.965-0.992	0.002	3.219	2.021-5.127	<0.001	7.52
QTcH	1.045	1.032-1.057	<0.001	1.031	1.016-1.047	<0.001	0.978	0.964-0.992	0.002	3.166	2.007-4.995	<0.001	8.19*
QTcR	1.045	1.033-1.058	<0.001	1.026	1.011-1.042	0.001	0.980	0.966-0.993	0.004	2.866	1.716-4.786	<0.001	1.27

Level of significance: In a 4 parameters analysis a Δ -2 LLR between models >7.81 is statistically significant at level p<0.050. The regression models containing QTcFri and QTcH are significant better predictors of 1-year non-cardiac mortality compared to QTcB. QTcFri is also significant better than QTcR.

* =p<0.050; ° =p<0.010

Abbreviations:

-2 LLR Δ QTcB: difference of -2 Log Likelihood Ratio compared to the model including QTcB; CI: confidence interval; HR: hazard ratio; QTc>ULN: QTc value above upper limit of normal; QTcB: QT correction with Bazett formula; QTcFri: QT correction with Fridericia formula; QTcFra: QT correction with Framingham formula; QTcH: QT correction with Hodges formula; QTcR: QT correction with Rautaharju formula.
